# Mutual Relationships of Nanoconfined Hexoses: Impacts on Hydrodynamic Radius and Anomeric Ratios

**DOI:** 10.1021/acs.langmuir.4c01826

**Published:** 2024-09-22

**Authors:** Mia R. Halliday, Samantha L. Miller, Christopher D. Gale, Jenna R. Deckard, Bridget L. Gourley, Nancy E. Levinger

**Affiliations:** † Department of Chemistry, Colorado State University, Fort Collins, Colorado 80523-1872, United States; ‡ Department of Chemistry and Biochemistry, DePauw University, Greencastle, Indiana 46135-0037, United States; § Department of Electrical and Computer Engineering, Colorado State University, Fort Collins, Colorado 80523, United States

## Abstract

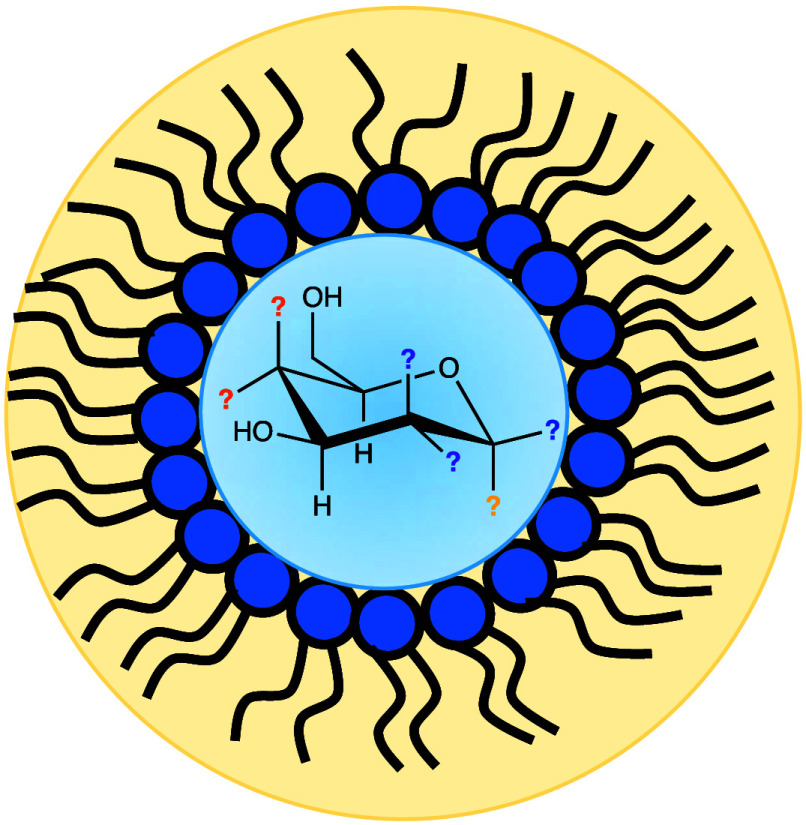

Although all hexose sugars share the same chemical formula, C_6_H_12_O_6_, subtle differences in their stereochemical structures lead to their various biological roles. Due to their prominent role in metabolism, hexose sugars are commonly found in nanoconfined environments. The complexity of authentic nanoconfined biological environments makes it challenging to study how confinement affects their behavior. Here, we present a study using a common model system, AOT reverse micelles, to study hexose sugars in nanoconfinement. We examine how reverse micelles affect the hexoses, how the hexoses affect reverse micelle formation, and the differences between specific hexoses: glucose, mannose, and galactose. We find that addition of glucose, mannose or galactose to reverse micelles that already contain water leaves their size smaller or nearly unchanged. Introducing aqueous hexose solution yields reverse micelles smaller than those prepared with the same volume of water. We use ^1^H NMR to show how the nanoconfined environment impacts hexose sugars’ anomeric ratios. Nanoconfined mannose and galactose display smaller changes in their anomeric ratios compared to glucose. These conclusions may provide insights about the biological roles of each hexose when studied under a more authentic nanoconfined system.

## Introduction

Hexose sugars are abundant throughout biology. The three most common hexose sugars are glucose, the fuel for our cells among other things, galactose, used in cell–cell signaling,^1,2^ and mannose, which is often added to proteins and lipids via glycosylation.^3,4^ Within biological systems, hexoses often perform these functions in crowded, nanoconfined environments within the cytoplasm of cells^5^ or in the crevices of proteins.^3,6^ The crowded, confined environments can affect hexose sugar characteristics, and, in return, the sugars can impact the crowded environments.^7−9^

**Figure 1:**
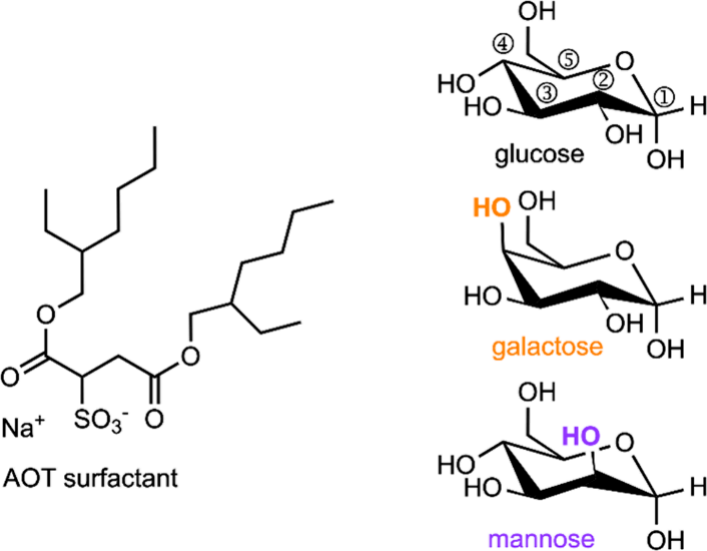
Chemical structures of (left) AOT surfactant and (right) representative α–-pyranose structures of d-glucose, d-mannose, and d-galactose. Purple and orange OH groups highlight stereochemical differences relative to glucose. Standard numbering is shown for glucose.

The nanoconfined environments of biology are exceptionally complex, so using a simple model system can provide an alternative environment for isolating size-dependent characteristics and behaviors. Here, we use reverse micelles to provide a size-tunable environment with which to study molecular interactions in confinement.^10,11^ A solution of reverse micelles contains a polar phase, usually water, a nonpolar phase such as isooctane, and a surfactant to stabilize the interface. Sodium bis(2-ethylhexyl) sulfosuccinate (also known as aerosol OT or AOT), whose structure appears in Figure 1, is perhaps the most common surfactant used to prepare reverse micelles due to the ease of their preparation and the excellent stability of the prepared emulsions.^10,12,13^ AOT-based reverse micelles have many desirable properties as a model system: they are easy to prepare, size tunable, and stable. Reverse micelles are typically characterized by the value , which has been shown to be proportional to the reverse micelle size.^14−22^ This unitless value is a ratio of concentrations and not an exact description of diameter. Often, reverse micelles are assumed to adopt a spherical shape, and on average this seems generally true, but simulations show that reverse micelles are aspherical on short time scales.^23−26^

Reverse micelles are well suited for encapsulating small osmolytes and biological metabolites, like hexoses.^7−9,27^ The Levinger group has explored the impact of reverse micellar confinement on various osmolytes^7−9,27^ including glucose.^7−9^ We have found that the confining environment slows down chemical exchange between water and glucose,^8^ raising the activation energy for proton exchange around room temperature but lowering the barrier at low temperatures.^9^ Glucose also changes the assembly of the AOT RMs.^7^ These studies spurred questions about how other hexose sugars behave in confinement.

In addition to naturally occurring d-hexoses and their unnatural l-hexose stereoisomers, hexoses can exist as eight different hexose stereoisomers, each with two anomeric states that all share the chemical formula, C_6_H_12_O_6_. Each hexose isomer can also exist in three forms: six-membered pyranose rings, five-membered furanose rings, or linear chains. Differences in stereochemistry lead to differences in the prevalence and biological activity of each form.^28^ We have focused on the three common d-isomers of glucose, mannose, and galactose because of their prevalence in biochemical systems. On the molecular level, glucose, mannose and galactose each have different stereochemical positioning of hydroxyl groups, as illustrated in Figure 1. Mannose, galactose and glucose—the hexoses studied here—exist almost entirely (>99%) in the pyranose form.^28,29^ Pyranose and furanose structures also exist either in the α or β form associated with the orientation of attachments to the anomeric carbon atom (position ① in Figure 1) where the −H and −OH groups can be positioned either axially and equatorially, or vice versa. When the –OH group is positioned axially, the structure is designated as an α anomer, illustrated in Figure 1. When the −OH group is positioned equatorially, the structure is designated as a β anomer. In the crystalline solid state, either the α or β form dominates, but in solution, both forms exist in equilibrium.^30,31^ This occurs because in solution, the anomers can interconvert via the linear intermediate, which has an achiral anomeric position.^28,32−34^

**Scheme 1:**
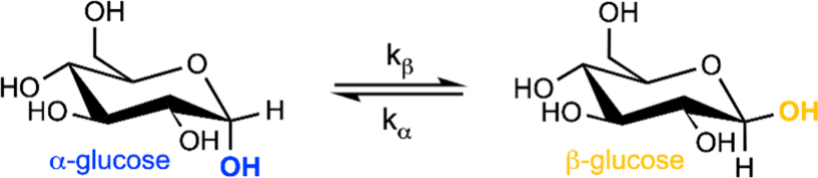
Anomeric Equilibrium between α (blue) and β (gold) Pyranose Forms of Glucose

The kinetics of the ring opening and closing reaction define the equilibrium between the anomers (Scheme 1) which in turn defines how much of each anomer is present in solution. Variables that may impact kinetics of this reaction have been reported, including solvent identity, and pressure.^28,30,32,33,35−37^ High polarity solvents can favor one anomer and while less polar solvents favor the other.^20,21^ Although, D_2_O can affect the individual forward and reverse rates, it does not change the equilibrium and ultimately the same anomeric ratio is reached.^22,23^ Interaction of hexoses with cations have also been reported to disrupt the normal anomeric ratio.^38−42^

Given the results we have observed for glucose within reverse micelles,^7−9^ here we explore the behavior of two additional common hexoses, mannose and galactose confined in AOT reverse micelles. We report how these hexoses impact reverse micelles and how the nanoconfinement affects the different hexose sugars. The addition of mannose and galactose broadens our study beyond glucose and allows us to study how sugars impact the reverse micelles and examine how the specific stereochemistry of the hexoses impacts their behavior within reverse micelles.

## Methods

Sodium bis(2-ethyl-hexylsulfosuccinate) (AOT ≥ 99%), D-galactose (anhydrous, 99%) and cyclohexane-d12 (99.5%) were purchased from Acros Organics (Waltham, MA) and used as received. 2,2,4-Trimethylpentane (isooctane, ≥ 99%, Sigma-Aldrich, St. Louis, MO), d-glucose (anhydrous, ACS grade, Fisher Scientific, Waltham, MA), D-mannose (anhydrous, 99%, Oakwood Chemical, Estill, SC), and deuterium oxide (D_2_O, 99%, Cambridge Isotope Laboratories, Andover, MA) were used as received. Millipore filtered and deionized water (18.2 MΩ-cm) was used to prepare reverse micelles. All glassware used was soaked in a 5 M nitric acid solution for at least 30 min, thoroughly rinsed with deionized (DI) water, and dried prior to use.

Hexose samples in bulk water were prepared by dissolving the hexoses in a solution of 90% water, 10% D_2_O to yield a 30:1, solvent:hexose mole ratio (∼1.9 M). Bulk D_2_O samples were prepared by dissolving the hexoses in D_2_O to yield the same concentration. Bulk aqueous samples discussed here constitute only the hexose in water, D_2_O, or a combination.

In a previous publication, we introduced two different ways that we prepare hexose containing reverse micelle samples: *hexose loaded* or *w*_0_*equivalent*,^7^ and we use these methods to prepare hexose containing reverse micelles here as well. Both preparations used a 0.1 M stock solution of AOT in isooctane. To prepare *hexose loaded* samples, we first added water to the AOT stock solution to form reverse micelles to a desired *w*_0_ value; then solid hexose was added to create the desired water:hexose ratio. To prepare *equivalent* samples, reverse micelles are formed with the addition of the *equivalent* volume of aqueous hexose solution, that would create the same *w*_0_ value if added as pure water. For example, to create a solution of reverse micelles at *w*_0_ = 10 containing only water, we added 180 μL of water to 10 mL of 0.1 M AOT in isooctane solution. To make a *hexose loaded w*_0_ = 10 reverse micelle solution, we subsequently added 0.0600 g (0.33 mmol) hexose to the water-only reverse micelle solution to yield a 30:1 water:hexose mole ratio in the RM. To make an *equivalent* sample, we prepared a 30:1 water:hexose solution and added 180 μL of this water/hexose solution to 10 mL of 0.1 M AOT in isooctane in place of water. Galactose *loaded* reverse micelles were not measured due to sample instability at any water:galactose ratio. A 30:1 ratio was chosen as it represents the highest concentration at which both *loaded* and *equivalent* reverse micelles remain stable over extended periods of time. To compare the two different preparation methods, we introduce a new parameter in the *Size measurements by Dynamic Light Scattering* section of the Results and Discussion.

Reverse micelle sizes were measured using dynamic light scattering (DLS, Malvern Zetasizer Nano ZS). Each size measurement comprises a series of 10 scans. We measured reverse micelles containing only water as well as glucose *equivalent*, glucose *loaded*, mannose *equivalent*, mannose *loaded* and galactose *equivalent* reverse micelles prepared as *w*_0_ = 10, 15, and 20. Each measurement was collected at 20 °C at a 172° backscattering angle. Samples were measured in a 1 cm path length glass cuvette. Measurement selectivity was based on a low polydispersity index (<0.3) to ensure particles were uniform in size. Analysis was performed using Zetasizer software (version 8.02). We report reverse micelle size based on number distribution to avoid skewing the distribution toward larger sized particles.

One dimensional ^1^H NMR spectra were collected using a Bruker Avance (Billerica, MA) spectrometer at 400 MHz with a cyclohexane d-12 lock solvent for reverse micelle samples and D_2_O for bulk samples. All spectra were acquired at standard conditions, 25 °C and 64 scans. Integration values were taken from OH peaks due to significant overlap between the upfield CH peaks and the water peak. These values were then compared to the literature value for bulk solution using a student *t* test to confirm statistical significance. Data processing, including baseline and phasing corrections, was done using MestReNova (version 14.1.2–25024).

## Results and Discussion

### Size Measurements by Dynamic Light Scattering (DLS)

**Figure 2:**
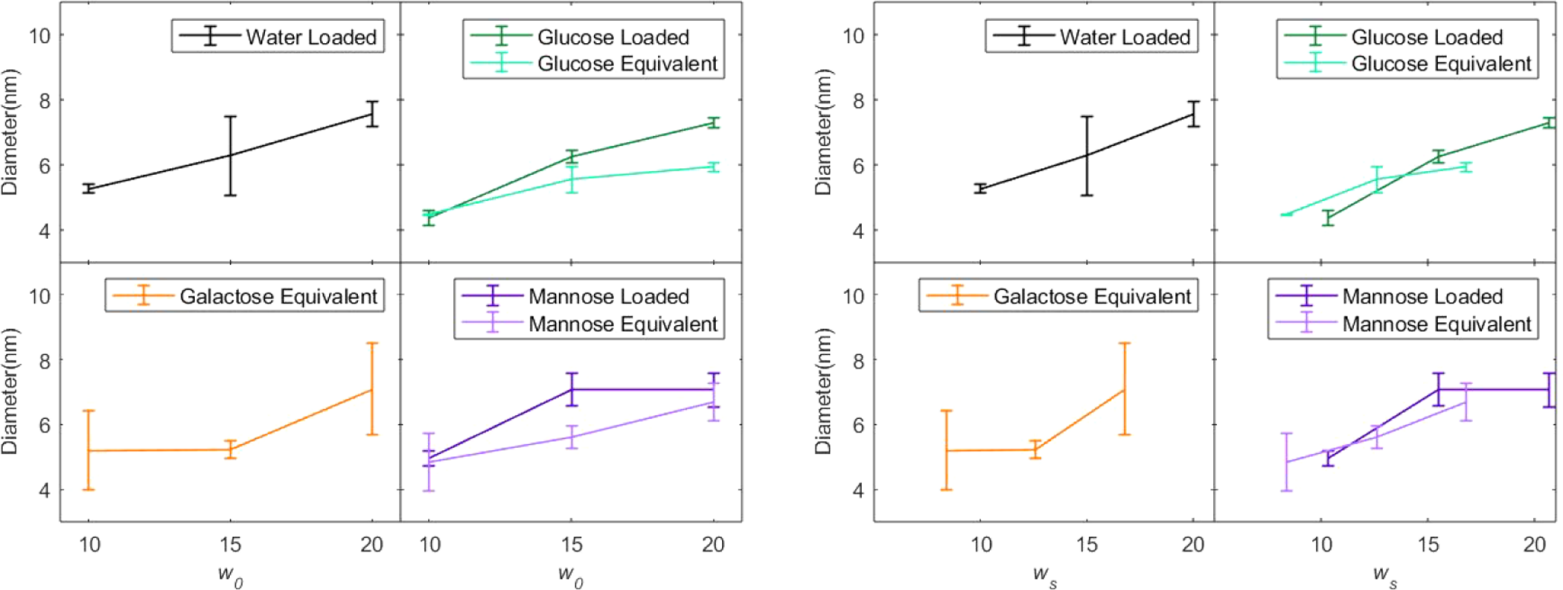
Left: reverse micelle diameter as a function of *w*_0_. Right: reverse micelle diameter as a function of *w*_*s*_. Reverse micelles were prepared as water only, glucose *loaded* and *equivalent*, galactose *equivalent*, or mannose *loaded* or *equivalent*. Error bars represent the standard deviation between 3 and 4 individual measurements of different samples.

The addition of hexose sugars impacts reverse micelle size. Because the hexoses are highly soluble in water and insoluble in the isooctane continuous phase, we might predict that adding hexose to a reverse micelle to form *hexose loaded* reverse micelles would increase the volume of the polar aqueous core. Likewise, it seems logical that the size of *equivalent* reverse micelles prepared with the same volume of polar solution as reverse micelles containing only water should yield reverse micelles with the same size as reverse micelles prepared with only water. However, this is not what we observe from DLS measurements shown in Figure 2. Graphs on the left side of Figure 2 plot the reverse micelle size as a function of *w*_0._ The sizes of *hexose loaded* reverse micelles, where hexose has been added to already formed reverse micelles containing water, are the same size or smaller than the pure-water reverse micelles of the same *w*_0_ value from which they were prepared. In contrast, *equivalent* reverse micelles, prepared with an equivalent amount of aqueous hexose solution, appear smaller than reverse micelles prepared with only water. We and others have observed this behavior previously for reverse micelles encapsulating glucose prepared either as *hexose loaded* or *equivalent*.^7,43^ Mannose and galactose demonstrate similar behavior, however, the magnitude of this effect differs depending on the specific hexose.

For reverse micelles containing only water, *w*_0_ serves as a proxy to describe reverse micelle size, because, for a given amount of water, the reverse micelle will always adopt a certain micelle size that is proportional to *w*_0_. However, *w*_0_ does not consider the effect of added solutes, making it insufficient to describe the size-dependent trends we observe for reverse micelles containing an additional solute, in this case, a hexose. We introduce a new parameter, *w*_s_, to describe the solute containing reverse micelles defined as,
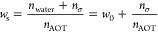
1where *n*_*i*_ is the moles of water (*n*_water_), AOT (*n*_AOT_), or solute (*n*_σ_) in the solution. In samples reported here the solute is d-glucose, d-mannose, or d-galactose. Table 1 reports the values for each sample prepared. Target *w*_0_ describes how samples were prepared, that is, with sufficient water or aqueous hexose solution to achieve a “target” *w*_0_ value. Although the ratio of water to hexose in the reverse micelles is constant for *hexose loaded* and *equivalent* preparations, the actual values of *w*_0_ and *w*_s_ differ depending on the method of preparation. For example, the value of *w*_0_ for *hexose loaded* reverse micelles is the same as the target *w*_0_ value while values of *w*_0_ and *w*_*s*_ for *equivalent* reverse micelles will always be lower than the target *w*_0_. Using *w*_s_ makes it possible for us to compare the reverse micelles regardless of how we have prepared them. Using the new *w*_s_ parameter therefore improves the specificity of the characterization by including contributions from all components in the polar core of the reverse micelle.

**Table 1:** Water and Hexose Parameters for Reverse Micelles Prepared^a^

	hexose loaded		equivalent^b^	
target *w*_0_	actual *w*_0_	*w*_s_	actual *w*_0_	*w*_s_
10	10	10.3	8.1	8.4
15	15	15.5	12.2	12.6
20	20	20.7	16.2	16.8

a Hexose loaded reverse micelles are prepared with water to which hexose is later added. Equivalent reverse micelles are prepared with a volume of aqueous hexose solution that would create the “equivalent” water only containing reverse micelle. All samples have a water:hexose ratio of 30:1.

b We used densities for aqueous hexose solutions reported by Zhuo et al. to determine water and hexose amounts in *equivalent* reverse micelles.^40^

The addition of hexose to the reverse micelles affects their size and DLS measurements show that this size depends on the solutes dissolved in the water pool, a key variable in our experiments. The sizes of AOT reverse micelles containing only water track remarkably well with *w*_0_.^22,44^ Graphs on the right side of Figure 2 show that when we compare hexose containing reverse micelles as a function of *w*_s_, the size of the reverse micelles scales approximately linearly with *w*_s_. The method of preparation does not appear to play a significant role in the behavior.

The impact of the hexose on reverse micelle size appears to depend on the specific hexose under consideration. We observe a more modest contraction of the reverse micelle size for mannose or galactose containing reverse micelles compared to the effect of glucose. We discuss this later in the paper in the section titled Analysis of Size Trends.

### Anomeric Ratios Determined by ^1^H NMR Spectroscopy

**Figure 3:**
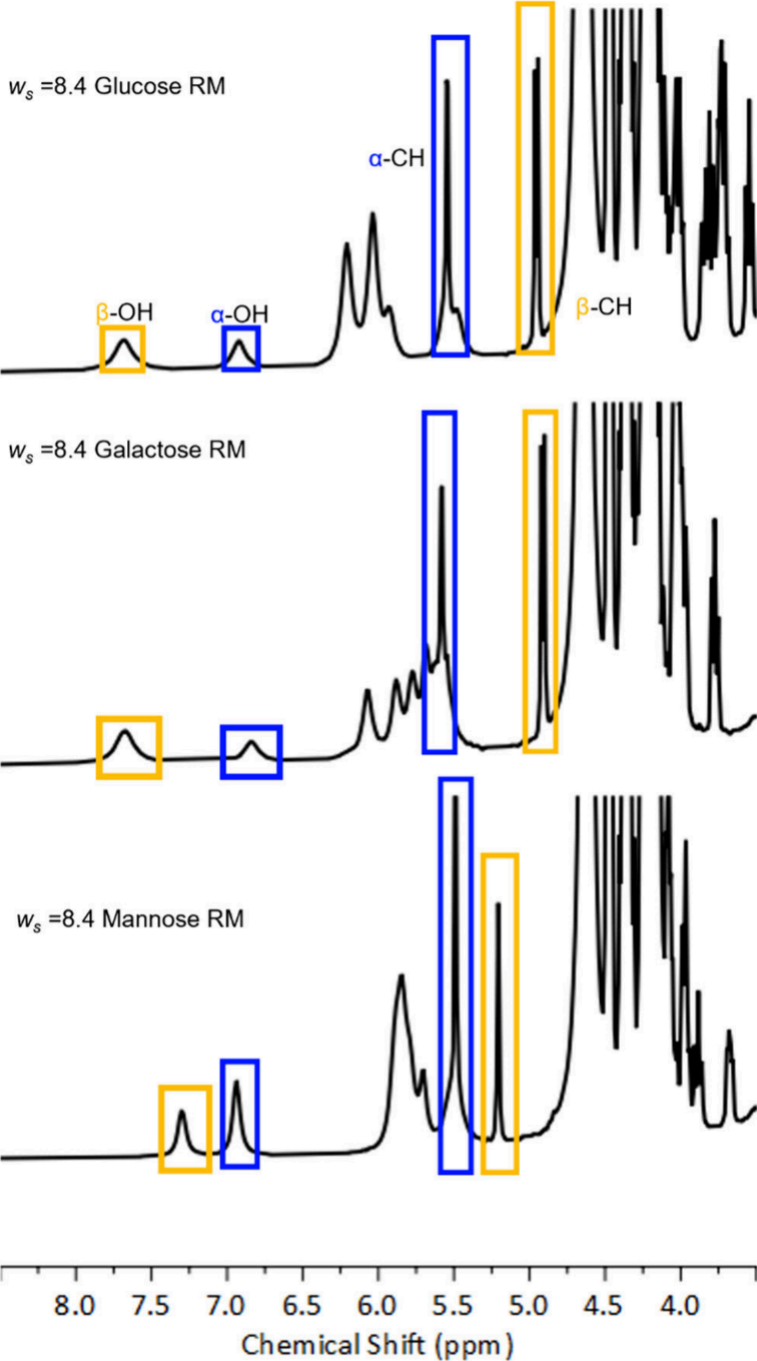
^1^H NMR spectra of AOT reverse micelles with *w*_s_ = 8.4 encapsulating glucose (top), galactose (middle), and mannose (bottom). Blue and gold boxes identify signals from α and β peaks, respectively.

**Figure 4:**
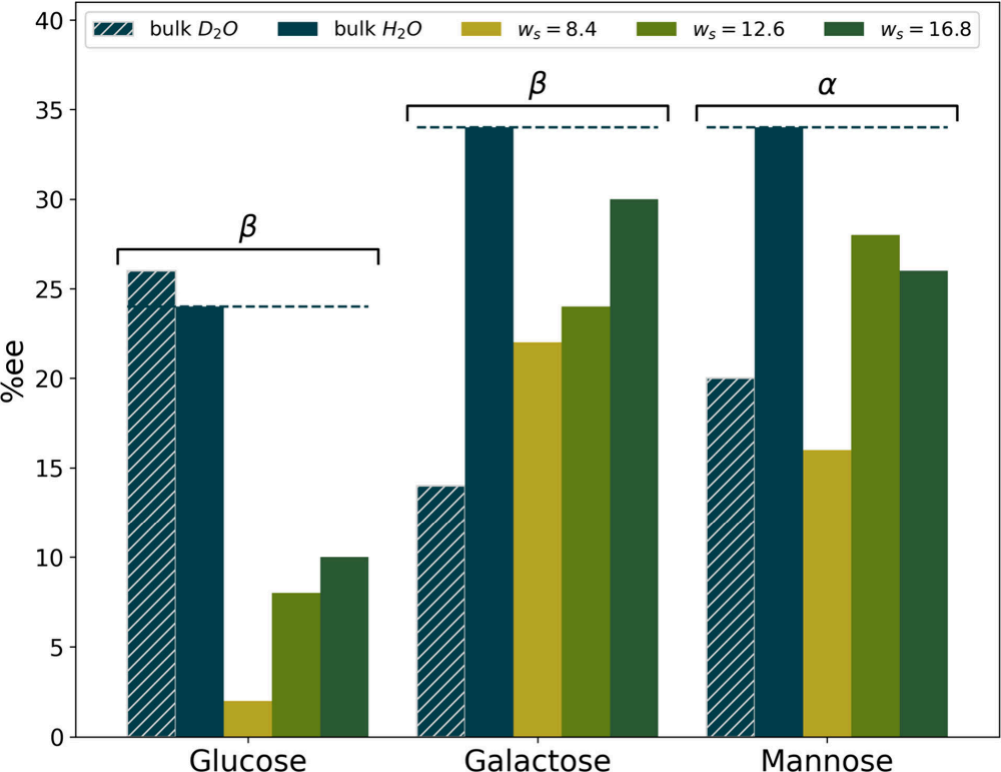
Comparison of the enantiomeric excess (% ee) as a function of the hexose and environment. A dashed line is provided at the % ee for the bulk H_2_O value to emphasize the change with nanoconfinement. Labels are added above each hexose to indicate whether the % ee indicates an excess of the α or β anomer. Uncertainties are too small to display and are listed in Table S2.

The ^1^H NMR spectroscopy demonstrates that the confined reverse micelle environment impacts the anomeric ratios of glucose, galactose, and mannose typically observed in bulk solution. In bulk aqueous solution, fast exchange with water broadens then coalesces the hexose α-OH and β-OH peaks with the dominant water peak, so the peaks are absent from the NMR spectra, and cannot be quantitatively integrated (Figure S1). However, the reverse micelle system slows this exchange, revealing well-resolved hydroxyl peaks that can be integrated to determine the anomeric ratio present in reverse micelles.^8^Figure 3 clearly shows α-OH and β-OH peaks in the ^1^H NMR spectra of galactose, glucose, and mannose in reverse micelles. Integration of the α-OH and β-OH peaks yields anomeric ratios, reported in Figure 4. We measure the anomeric ratios for bulk aqueous solutions from the α-CH and β-CH peaks (spectra shown in Figure S1), the α-OH and β-OH peaks are so small that it is not possible to get a quantitative measure of the peak area.

From the NMR spectra we calculate the enantiomeric excess (% ee) of each hexose. By integrating the area under the α-OH and β -OH peaks in each spectrum, we determine the relative concentration of each anomer, then determine the % ee by
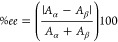
2where *A*_α_ and *A*_β_ represents the integrated area of the α- and β-OH peak in the NMR spectrum, respectively. Values for the relative area of each peak and the resulting % ee are given in Table S2. Figure 4 provides a comparison of the % ee in reverse micelles and in bulk aqueous solution. In bulk aqueous solution, our spectra show both glucose and galactose distributions display approximately one-third α and two-thirds β, consistent with published data,^33,36,45^ while the mannose distribution displays the reverse with approximately two-thirds α and one-third β anomer.^33,36,45^ In reverse micelles, we observe a consistent drop in % ee, indicating a trend toward a 50:50 ratio between anomers. This effect is largest in *w*_s_ = 8.4 reverse micelles. As the reverse micelle size increases, the anomeric distribution slowly approaches the anomer ratio seen in bulk aqueous solution, although it does not become fully bulk-like, even at *w*_s_ = 16.8, the highest value measured.

### Analysis of Size Trends

The observed trends in size point to an interaction of the hexose sugars with surfactant’s sodium counterions at the inner aqueous reverse micelle interface. We have previously suggested the trend in reverse micelle size could arise from interactions between AOT and glucose,^7^ which remains in agreement with results presented here. We hypothesize that the position of the hexose molecule within the reverse micelle interior affects the size of the reverse micelle. When hexoses reside near the edge of the water pool, the anomeric ratios observed in Figure 4 demonstrate that they likely interact strongly with the Na^+^ counterion.^31,38,40,46^ This close interaction will partially screen the charge. As the AOT experiences less of the balancing counterion Na^+^ cation’s charge, it will experience increased Coulombic repulsion between AOT headgroups, corresponding to an increase in the surface area of each AOT molecule, increasing the total surface area in solution. For a fixed volume of polar material, the only ways to increase the surface area are to either create more, smaller reverse micelles, or to change the shape.^7,24^ However, the data presented in Figure 2 cannot determine which factor is changing the apparent size of the reverse micelles to accommodate this additional surface area.

Other factors could impact the observed reverse micelle size. In a previous publication, we considered whether changes to glucose partial molar volume or particle shape could explain the changes in reverse micelle sizes we observe.^7^ Although negative partial molar volumes of glucose, galactose and mannose have been observed, the magnitude of the contraction is approximately 5%.^47−50^ With a molar ratio of 30:1 water:hexose, this contraction is too small to account for changes in reverse micelle size that we observe. As previously noted, the addition of hexoses to the reverse micelles could also cause the reverse micelle shape to change. In our previous report,^7^ we noted that even a substantial change of shape cannot completely account for the observed changes in reverse micelle size. In all likelihood, each of these factors, that is, a small negative partial molar volume, a change in the shape, and a change in the number and volume of reverse micelles, all contribute somewhat to the observed changes. Overall though, Figure 2 demonstrates that the most dominant factor controlling the size of the reverse micelles is the amount of polar material enclosed, which is captured well by w_s_, as compared to the apparent misconceptions created by w_0_.

### Analysis of Changes in Anomeric Ratio

The anomeric ratios characteristic of the hexose isomers are typically explained by the decreased steric strain placed on the ring structure when the OH groups are in the equatorial positions are filled. It seems unlikely that confinement in a space, however small, could reduce the steric strain of the minor anomer to such a degree as to match the relative stability of the major anomer. The clear, size-dependent change to the anomeric ratios of all hexoses tested suggests not just a general interaction, but a specific interaction that only occurs when the hexose is forced to interact by the small size of the reverse micelle.

We propose that the reverse micelle’s effect on hexose anomer ratio can be explained by the formation of a novel hexose-Na^+^ complex. Complexes between hexoses and cations are not new and were first discovered by paper electrophoresis, where researchers found that different hexoses could be separated when mixed with certain salts and subjected to an electric field.^31^ Researchers have found that certain sugars form complexes with cations. Specifically, a pattern of hydroxyl groups arranged in an axial–equatorial-axial configuration can lead to hexose-cation complexes, particularly for highly charged cations, such as calcium.^31,38,40,46,51,52^ The binding is typically very weak; for example calcium gives a typical binding constant of *K*_eq_ ∼ 3 M^–1^.^52^ In the case presented here the complex forms with a Na^+^ cation, so the interactions should be even weaker compared to calcium. Measurements of glucose in bulk aqueous NaCl solution measured by polarimetry and predicted by quantum chemical and molecular dynamics calculations show no preference for Na^+^ interacting with either anomer.^41^ However, we hypothesize that confinement to the reverse micelle interior could lead to Na^+^ complexes with hexoses and explain the changes to the anomeric ratio reported in Figure 4.

**Scheme 2:**
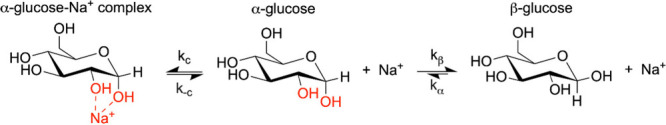
Proposed Competing Equilibria between Anomeric and Na^+^ Complex Formation With Glucose Hydroxyl groups shown in red can form the proposed complex with the α anomer of glucose.

This axial–equatorial-axial hydroxyl pattern does not exist in any of the hexoses we measured, regardless of anomer. To explain the trends we observe, we propose that confinement in the reverse micelle can lead to a complex between the AOT Na^+^ counterion with just two hexose hydroxyls arranged in an axial–equatorial pattern. All three of the hexoses we measured would have at least one site where complexation with just two hexose hydroxyl groups can occur for one of the anomers. This occurs because regardless of the stereochemistry of the hydroxyl at the ② position of the hexose, both anomers are present and so one of them is guaranteed to form an axial–equatorial arrangement with the ② position. Formation of a complex with Na^+^ adds a new reaction to the typical equilibrium for hexose anomerization, creating a situation where one anomer has an additional equilibrium between the complexed and noncomplexed states that competes with the anomerization reaction. Scheme 2 shows this series of equilibria we expect for glucose in AOT reverse micelles. The complexed state will not undergo anomerization without first dissociating from the Na^+^, thereby effectively removing the complexed population from the anomer equilibrium. This causes the equilibrium of uncomplexed sugars to shift toward the complexing anomer. For glucose and galactose, which have an equatorial hydroxyl group at the ② position, the α anomer generates the axial–equatorial arrangement that would be necessary for complexation, while for mannose it is the β anomer that forms the axial–equatorial arrangement. In all three cases, the complex forms with the minor anomer, causing an increase in the minor anomer and a drop in % ee.

**Scheme 3:**
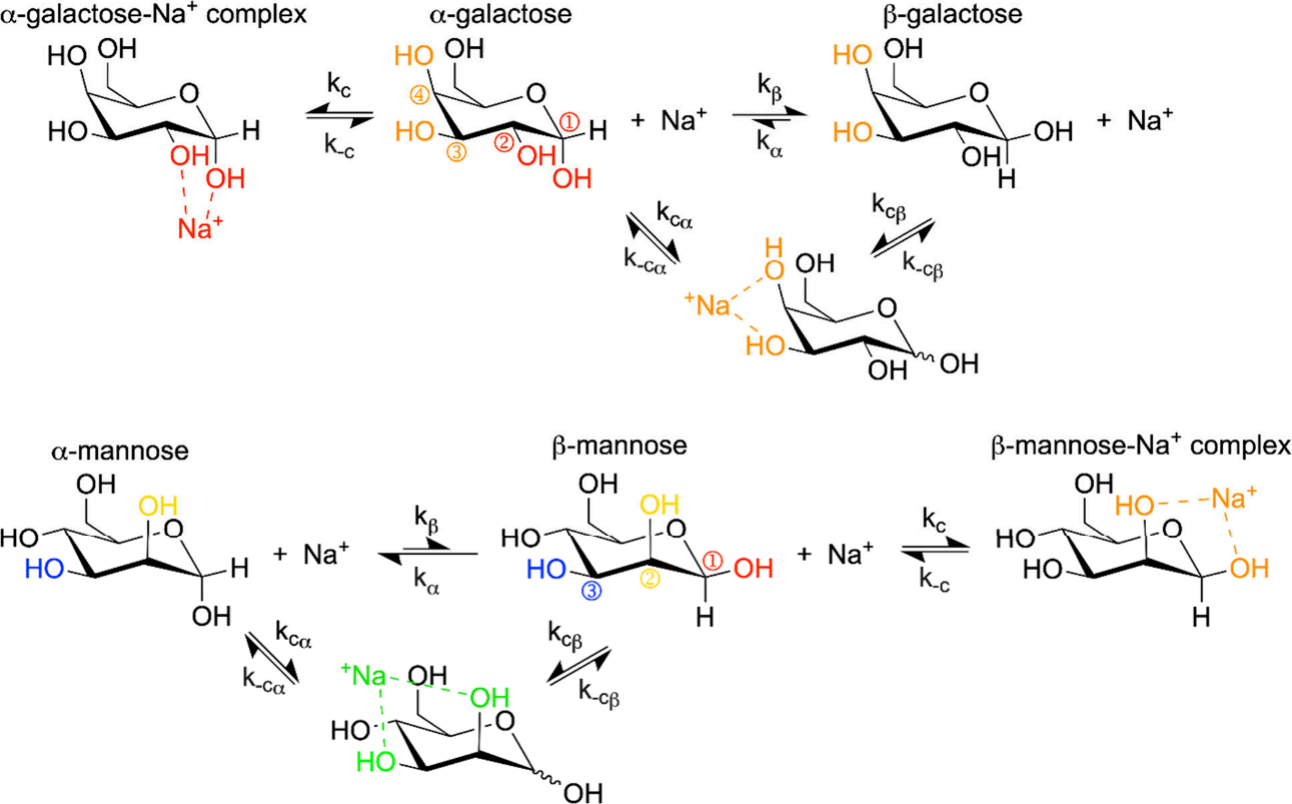
Proposed Competing Equilibria for Galactose and Mannose For galactose (top), hydroxyl groups in ①-② positions shown in red form the proposed complex of Na^+^ with only the α anomer while orange hydroxyl groups in ③-④ positions complex with either anomer. For mannose (bottom), red and yellow hydroxyl groups in ①-② positions form the proposed complex of Na^+^ with only the β anomer while yellow and blue hydroxyl groups in ②-③ positions complex with either anomer.

A complex formed between hexoses and Na^+^ in reverse micelles can also explain the differences we observe between specific hexoses. Because we are only observing the ratio between anomers via ^1^H NMR, Figure 4 is only sensitive to complexation with Na^+^ involving the anomer hydroxyl group, but galactose and mannose also present the axial–equatorial arrangement of hydroxyls elsewhere in the molecule, between positions ③ and ④ for galactose and positions ② and ③ for mannose as shown in Scheme 3. The change in % ee between bulk hexose solution and hexose in reverse micelles should be roughly proportional to the degree of complexation with Na^+^ specifically between the ①-② position hydroxyl groups. Because there is a second binding site in galactose and mannose, the second site competitively inhibits the formation of the complex at the ①-② positions, reducing the effect on the enantiomeric excess. This explains why the enantiomeric excesses measured for galactose and mannose are less impacted by nanoconfinement than glucose is. It is also worth noting that due to the off -center nature of the interaction between the ①-② positions and Na^+^, the hexose is naturally oriented so that it can conform at least somewhat to the curvature of the interface. The ③-④ positions where galactose can bind Na^+^ are in a similar position on the opposite side of the ring and should also orient the hexose to follow the curvature, but the ②-③ positions found in mannose are in between, which could cause a less favorable orientation at the interface and reduce the binding constant at the ②-③ positions. This would weaken the competitive inhibition with the anomeric, ①-② positions binding site and explain why mannose has a slightly larger change in the % ee compared to galactose.

Although complexes of galactose, or mannose with Na^+^ have not been directly observed, all previous studies present results only from bulk solution. The reverse micelles in our study facilitate the complex formation in two ways. The Na^+^ ions in reverse micelles are arranged around the interface rather than freely mixing into the aqueous interior,^25,26,53−56^ which means that the local Na^+^ concentration is significantly higher than simply the moles of Na^+^ per reverse micelle divided by the average volume of the reverse micelle. Second, for the same reason that Na^+^ occupies the interface rather than the interior of the water pool, solutes often occupy the reverse micelle interfacial region more often than the interior, especially as *w*_s_ decreases, likely an example of a nanoconfinement-induced hydrophobic effect.^7,57,58^ Taken together, these factors should push hexoses to favor locations at the interface and then drive complex formation with the excessive concentration of sodium cations at the interface, making structures that have never been observed in bulk solution suddenly possible in AOT reverse micelles.

We considered another mechanism that could account for the disruption in the hexose anomeric ratios. If the reverse micelle environment enhanced hexose–hexose interactions, the confining environment could lead to dimer or larger aggregate formation. For each hexose, aggregation should favor the less soluble anomer and remove the aggregated species from the mutarotation equilibrium and shift the anomeric ratio. Although this mechanism should disrupt the anomeric ratio in the direction we observe, it cannot account for changes in the reverse micelle sizes as the cation interactions do. Additionally, given that galactose and mannose are less water-soluble than glucose, we would expect this mechanism would occur more readily for them than glucose, the opposite of what we observe. Thus, this mechanism does not provide an adequate explanation of our results.

It is important to note that these systems are not simple and clean in almost any sense of the word. Our recent work has demonstrated that AOT reverse micelles are not spherical,^24^ there is no understanding of how the hexoses impact the shape beyond the simple argument that they likely do impact the shape, as shown in Analysis of Size Trends. The shape would have a dramatic impact on properties like the hexose’s distance to the interface. Because the complex we believe is responsible for the change in anomeric ratio depends on the exceptionally high local concentration of Na^+^ at the interface, the shape could also play a key role in the anomeric ratios observed and explain some of the minor variations between hexoses observed. It is tempting to determine a representative stoichiometry for a given reverse micelle and spheres are convenient and useful for this purpose, but they also minimize the surface-area-to-volume ratio of the particle. Any other shape would change this property and the amount it changes will also depend on the specific shape the reverse micelle adopts. This makes what seems like a simple and useful heuristic remarkably speculative, so we refrain from providing more concrete examples of the number of hexoses per reverse micelle and their proximity to the interface.

## Conclusions

The stereochemistry of hexose sugars often impacts just how those sugars behave in solution. Not only that, but under nanoconfined conditions, glucose, the most common of the hexoses, is known to be impacted. Here we show that the interactions known to occur between glucose and AOT reverse micelles are also present between both mannose and galactose. When solvated in the water pool of a reverse micelle, we find that the sugars investigated here decrease the size of the reverse micelle, as compared to what would be expected for the volume of a similarly sized water-pool. The magnitude of this effect changes with the identity of the hexose. These results are consistent with interactions between AOT-Na^+^ counterions and the hexose sugars. In smaller reverse micelles, the sugars are hypothesized to exist in between the counterions and the AOT headgroup, on the boundary of the water pool, shielding the headgroups from the counterions and increasing Coulombic repulsion between the headgroups.

This would increase the surface area of the reverse micelle, explaining the size data presented. We have also demonstrated how the location of hexoses at the inner surface of the reverse micelle can disrupt the standard enantiomeric excess observed for the glucose, galactose and mannose anomers. NMR spectra reveal that the confined environment leads to a shift in the aqueous equilibrium toward the minor anomer that we attribute to the formation of Na^+^-hexose complexes. Differences between the three hexoses studied–glucose, galactose, and mannose–demonstrate subtleties of these important molecules where complexation with Na^+^ impacts both anomeric ratios and reverse micelle sizes. The NMR spectral data taken in conjunction with reverse micelle size data support the idea that hexose sugars reside near the boundary of the water-pool and the AOT.

The results presented have significant implications in a wide range of fields. Hexose sugars are often found in biology residing in nanoconfined environments. The results presented here suggest potential for confinement to affect anomeric ratios, which could impact their biological function. Knowing the interactions between the environment and the hexose sugars has implications in pharmaceuticals, protein mechanism research, and many other fields.

## Supporting Information Available

Table S1, sizes of AOT reverse micelles expressed as the hydrodynamic diameter in nm; Figure S1, ^1^H NMR spectra showing appearance lack of peaks from 6.0 to 8.0 ppm in water loaded reverse micelles and solutions of 2 M hexose in D_2_O; Table S2, anomeric ratios of glucose, mannose, and galactose in reverse micelles (PDF)
