# Supplementary material for: Mutual Relationships of Nanoconfined Hexoses: Impacts on Hydrodynamic Radius and Anomeric Ratios
Source: Langmuir. 2024 Sep 22;40(40):20918–26. doi: 10.1021/acs.langmuir.4c01826 (PMC11468786; doi:10.1021/acs.langmuir.4c01826)
Supplement: Supplementary file 1 — la4c01826_si_001.pdf [file la4c01826_si_001.pdf]

Mutual Relationships of Nanoconfined Hexoses: Impacts on Hydrodynamic Radius and Anomeric Ratios

Mia R. Halliday[a], Samantha L. Miller[a], Christopher D. Gale[a], Jenna R. Deckard[b], Bridget L. Gourley[b], and Nancy E. Levinger[a,c,*]

[a] Department of Chemistry, Colorado State University, Fort Collins, CO 80523-1872

[b] Department of Chemistry and Biochemistry, DePauw University, Greencastle, IN 46135-0037

[c] Department of Electrical and Computer Engineering, Colorado State University, Fort Collins, CO 80523

Supporting Information:

The dataset from this article and its supporting information can be found in the Dryad repository at DOI: 10.5061/dryad.h9w0vt4sc.

Table S1. Sizes of AOT reverse micelles from dynamic light scattering experiments. Sizes are expressed as the hydrodynamic diameter in nm. Values represent an average of 3-4 measurements. Error was calculated as the standard deviation between measurements.

w_s[a]	Water	Glucose	Mannose	Galactose
8.4		4.47 ± 0.03	4.83 ± 0.89	5.20 ± 1.22
10	5.26 ± 0.14			
10.3		4.36 ± 0.24	4.95 ± 0.22	
12.6		5.54 ± 0.42	5.61 ± 0.34	5.23 ± 0.28
15	6.28 ± 1.21			
15.5		6.25 ± 0.19	7.08 ± 1.05	
16.8		5.93 ± 0.13	6.69 ± 0.58	7.08 ± 1.41
20	7.57 ± 0.39			
20.3		7.30 ± 0.15	7.07 ± 0.52	

a For water containing reverse micelles, $w_s = w_0$.

Based on published aggregation numbers for AOT reverse micelles and a 30:1 water:hexose ratio, we estimate that each reverse micelle contains several hexose molecules. However, a geometric estimate indicates that there is ample room to solvate all these molecules in the reverse micelle interior. Specifically, from published values for the molar volume of glucose and mannose,[1] we find the molecular volume for each hexose is:

$$V_2^o = 112 mL/mol = 0.186 nm^3/molecule$$

Assuming a spherical reverse micelle, as a general approximation, and using the sizes listed in Table S1, the reverse micelle volume is at least 100 times larger than the hexoses.

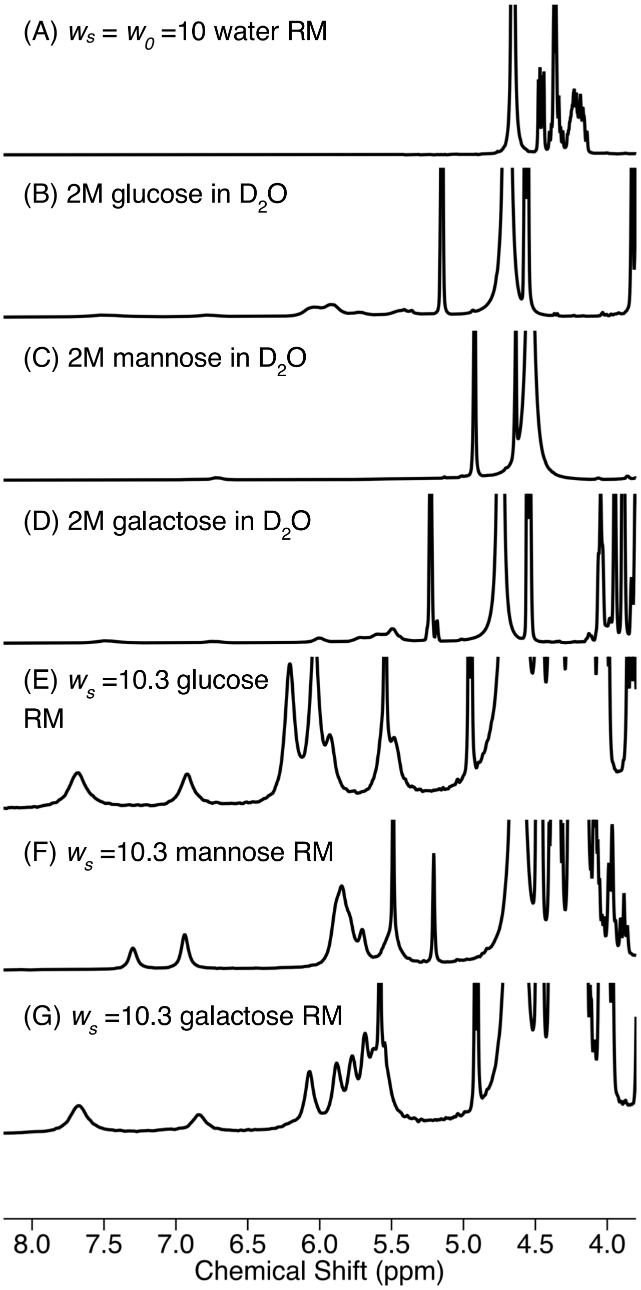

Figure S1. Representative NMR spectra of reverse micellar and aqueous solutions of showing the chemical shift range where OH peaks appear. (A) w_s=10.3 AOT reverse micelle containing only water; (B)-(D) hexose in bulk aqueous (D_2O) solution; (E)-(G) w_s=10.3 AOT reverse micelle containing 30 water:1 hexose as labeled in the legend. Reverse micelle samples clearly show peaks associated with anomeric OH groups in the 6.0-8.0 ppm range while these peaks are absent in bulk solutions of 2M hexose in D_2O.

Table S2. Average anomeric percentages of hexoses in AOT reverse micelles and bulk aqueous solution. Uncertainty represents the standard deviation between measurements of 3-4 individual samples.

	Glucose		Mannose		Galactose[a]	
w_s	$A_α$ (%)	$A_β$ (%)	$A_α$ (%)	$A_β$ (%)	$A_α$ (%)	$A_β$ (%)
8.4	49± 0.04	51± 0.04	58± 0.06	42± 0.06	39± 0.05	61± 0.05
12.6	46± 0.02	54± 0.02	64± 0.01	36± 0.01	38± 0.02	62± 0.02
16.8	45± 0.01	55± 0.01	63± 0.02	37± 0.02	35± 0.01	65± 0.01
Bulk[b]	38± 0.05	62± 0.05	67± 0.04	33± 0.05	33± 0.2	67± 0.2
2M D_2O[c]	37	63	60	40	43	57
	38	62	-	-	-	-

a OH peaks were integrated due to significant baseline overlap of CH peaks and water peak for w_0=15 and 20.

b Values taken from Zhu, *et al.*[2]

c Confirmatory measures of anomeric percentages for D_2O were performed for comparison with literature values for H_2O.

Reference:

(1) Fucaloro, A. F.; Pu, Y.; Cha, K.; Williams, A.; Conrad, K. Partial Molar Volumes and Refractions of Aqueous Solutions of Fructose, Glucose, Mannose, and Sucrose at 15.00, 20.00, and 25.00°C. *Journal of Solution Chemistry*, 2007, *36*, 61–80. https://doi.org/10.1007/s10953-006-9100-7.

(2) Zhu, Y. P.; Zajicek, J.; Serianni, A. S. Acyclic Forms of 1-C-13 Aldohexoses in Aqueous Solution: Quantitation by C-13 NMR and Deuterium Isotope Effects on Tautomeric Equilibria. *Journal of Organic Chemistry* **2001**, *66* (19), 6244–6251. https://doi.org/10.1021/jo010541m.
